# A common variant of the *NOTCH4* gene modulates functional connectivity of the occipital cortex and its relationship with schizotypal traits

**DOI:** 10.1186/s12888-020-02773-z

**Published:** 2020-07-09

**Authors:** Xiaohui Xie, Meidan Zu, Long Zhang, Tongjian Bai, Ling Wei, Wanling Huang, Gong-Jun Ji, Bensheng Qiu, Panpan Hu, Yanghua Tian, Kai Wang

**Affiliations:** 1 Department of Neurology, The First Affiliated Hospital of Anhui Medical University, Hefei, 230022 China; 2 Collaborative Innovation Center for Neuropsychiatric Disorders and Mental Health, Anhui Medical University, Hefei, 230022 China; 3 Anhui Province Key Laboratory of Cognition and Neuropsychiatric Disorders, Hefei, 230022 China; 4 Department of Medical Psychology, Chaohu Clinical Medical College, Anhui Medical University, Hefei, China; 5 Center for Biomedical Engineering, University of Science and Technology of China, Hefei, Anhui China

**Keywords:** *NOTCH4*, rs204993, Functional connectivity strength, Occipital cortex, Schizotypal traits

## Abstract

**Background:**

Schizotypal traits are considered as inheritable traits and the endophenotype for schizophrenia. A common variant in the *NOTCH4* gene, rs204993, has been linked with schizophrenia, but the neural underpinnings are largely unknown.

**Methods:**

In present study, we compared the differences of brain functions between different genotypes of rs204993 and its relationship with schizotypal traits among 402 Chinese Han healthy volunteers. The brain function was evaluated with functional connectivity strength (FCS) using the resting-state functional magnetic resonance image(rs-fMRI). The schizotypal traits were measured by the schizotypal personality questionnaire (SPQ).

**Results:**

Our results showed that carriers with the AA genotype showed reduced FCS in the left occipital cortex when compared with carriers with the AG and GG genotypes, and the carriers with the AG genotype showed reduced FCS in the left occipital cortex when compared with carriers with the GG genotype. The FCS values in the left occipital lobe were negatively associated with the SPQ scores and its subscale scores within the carriers with the GG genotype, but not within the carriers with AA or AG genotype.

**Conclusion:**

Our results suggested that the common variant in the *NOTCH4* gene, rs204993, modulates the function of the occipital cortex, which may contribute to schizotypal traits. These findings provide insight for genetic effects on schizotypal traits and its potential neural substrate.

## Background

Schizophrenia is a chronically severe, mental disorder, affecting approximately 1% of the worldwide population [1]. In addition, some individuals present preclinical symptoms of schizophrenia called schizotypal traits [2]. Schizotypal traits refer to a set of traits, continually distributed in the general population, that resemble some of the symptoms of schizophrenia. Schizotypal traits provide important insights into understanding the origins of schizophrenia [3]. Raine et al. concluded that structural and functional alterations in the brain induced by genetic and early environmental influences contribute to the development of schizotypy [4]. Indeed, schizotypal traits are more frequent in first-degree relatives of patients with schizophrenia, suggesting a genetic link between schizotypal traits and schizophrenia [5, 6]. The schizophrenia-risk gene also contributes to the presence of schizotypal traits [7, 8]. Hence, the schizophrenia-risk gene may have potential modulatory effects on schizotypal traits.

Although previous studies have found that some genes indicate susceptibility for schizophrenia [9, 10], only a small percentage of the genes have been consistently studied among different racial populations. The *NOTCH4* (neurogenic locus notch homolog protein4) gene, located in the major histocompatibility complex (MHC) region of 6p21.3 in humans, is highly associated with schizophrenia among different racial populations [11–13]. Wei and Hemmings found that the *NOTCH4* locus was involved in susceptibility to schizophrenia in a British population [13]. The relationship between schizophrenia and the common variants of *NOTCH4* gene was also found among different racial populations (the rs2071287 and rs3131296 in European and Japanese population) [14, 15]. However, these SNPs were not confirmed among Chinese populations. Recently, a study among 218 Taiwanese families reported another SNP, rs204993 with a significant relationship with schizophrenia, found by testing the association of the entire genomic region of *NOTCH4* [16]. Specially, the AA genotype of rs204993 was associated with a higher risk for schizophrenia in the Chinese Han population [17]. These findings suggested the common variant of the NOTCH4 gene, SNP rs204993 may be a potential genetic basis for schizophrenia in the Chinese population.

Recent evidences suggested the intermediary role of brain function for the genetic effect on schizophrenia or schizotypal traits. However, the effect of the common variant of the NOTCH4 gene, SNP rs204993, on brain function and its relationship with schizophrenia trait has not been investigated. Imaging genetics may provide a neural basis to explain how the gene affects the occurrence and development of diseases via connecting the link between gene and brain diseases [18]. Resting-state functional magnetic resonance imaging (rsfMRI), as a task-free and noninvasive measurement tool, has been used in several mental disorders [19]. Functional connectivity strength (FCS), which identifies functional hubs of human brain networks based on the graph theory method [20], is a developed method to test the connectivity of each voxel with all other voxels in the brain [21]. The FCS analysis has been used to characterize the neural mechanism in various psychiatric disorders [22], including schizophrenia and schizotypal traits [23, 24]. For example, decreased FCS in the bilateral occipital cortex and the right sensorimotor cortex as well as increased FCS in the bilateral temporal cortex, hippocampus, and the left prefrontal cortex were observed in schizophrenia [21]. Moreover, schizotypal traits presented abnormal connectivity in the default mode network compared with healthy controls [24].

In present study, we aimed to investigate the genetic effect of the schizophrenia-related gene (*NOTCH4* rs204993 genotype) on brain function and its relationship with schizotypal traits among healthy volunteers. Based on the above findings, we propose that the schizophrenia-risk rs204993 genotype (AA genotype) may be linked with attenuated function in schizophrenia-related brain regions, which contributes to schizotypal traits.

## Methods

### Participants

We recruited 641eligible healthy Chinese Han participants from the Anhui Medical University in Hefei, Anhui province. The inclusion criteria included: 1) undergraduate students; 2) the participant and whose parents were the Chinese Han populations; 3) age 18–25 years old; 4) Voluntary to be taken blood sample and MRI scanning; 5) no contraindication for MRI scanning. Exclusion criteria included: Psychiatric disorders, neurological diseases, alcohol or drug abuse, brain injury, visible brain structure abnormities, or excessive motion during MRI scanning (> 2.0 mm, 2.0°). Participants with a first-degree relative with a neurological or psychiatric disorder were also excluded. Of these participants, a total of 239 participants were excluded because of *NOTCH4* genotyping failure (12 participants) or poor MRI data (198 participants with excessive head motion and 29 participants with artifact in their structural images). Finally, a total of 402 participants were included in the data analysis of the current study. The study was approved by the Anhui Medical University Ethics Committee, and all participants signed informed consent forms after being given a thorough description of the study.

### SNP genotyping

Blood samples were collected to obtain DNA using standard procedures. The genotyping of all participants was determined in comparison with control DNA confirmed by sequencing in the SNP pattern. We used the Light Scanner Primer Design software program (Idaho Technology, UT, USA) to design the primer. The PCR primers were designed to amplify the desired locus, within which the target SNP (rs204993 in *NOTCH4* gene) is located: 5′-TTCGGGACTTCTGTTCAGCC-3′ (forward), 5′-TCCTGGAAGCACTCGTTGAC-3′ (reverse). Then, the amplicon library was used for high-throughput sequencing by Illumina HiSeq X Ten (Illumina, San Diego, CA, USA). Finally, the sequence reads were mapped to the human reference genome GRCH37 and the genotype of NOTCH4 rs204993 was divided into the AA, GA, and GG genotypes.

### Schizotypal trait evaluations

The schizotypal traits were evaluated using the schizotypal personality questionnaire (SPQ) [25]. The SPQ includes a 74-item with a “yes/no” response to assess schizotypal traits in the general population. The questionnaire subscale scores reflect different schizotypal dimensional features, including interpersonal, cognitive perceptual, and disorganized factors [26]. In the present study, we used the Chinese version SPQ, which has good internal consistency (0.95) and reliability value (0.86) for the Chinese population [27].

### Image data acquisition

Structural and functional MRI images of participants were acquired under a 3-T scanner at the University of Science and Technology of China, Anhui Province. Before images acquisition, all participants were asked to keep eyes closed and body still, and not to think of anything particularly. T1-weighted anatomical images with were acquired in sagittal orientation (TR/TE = 8.16/3.18 ms; flip angle = 12°; field of view (FOV) = 256 × 256 mm^2^;voxel size = 1 × 1 × 1 mm^3^; slice thickness = 1 mm; 188 slices). Functional MRI (BOLD) images were composed of 217 echo-planar imaging volumes (TR/TE = 2400/30 ms; flip angle = 90°; FOV = 192 × 192 mm^2^; voxel size = 3 × 3 × 3 mm^3^; slice thickness = 3 mm; matrix size = 64 × 64; and 46 continuous slices).

### Functional image preprocessing

The Data Processing Assistant for Resting-State Functional MRI imaging toolkit (DPARSF) was used for the functional image preprocessing, which was based on the following software: the Resting State Functional MR Imaging Toolkit and statistical parametric mapping software package (SPM8) [28, 29]. We discarded the first five volumes of data to ensure stable longitudinal magnetization, and the remaining volumes were corrected for slice timing and head motion. Next, we normalized the structural T1 image to the Montreal Neurological Institute space based on the T1 image unified segmentation with a 12 parameter nonlinear transformation. Finally, the data were nuisance regressed with 24 Friston motion parameters, white matter high signals, cerebrospinal fluid signals, and global signals as regressors, and filtered with a temporal band-pass of 0.01–0.1 Hz.

### Whole brain FCS analysis

A voxel-wise, whole-brain functional connectivity analysis was performed on the preprocessed rsfMRI data as in our previous study [30]. First, Pearson’s correlations between the residual time series of all pairs of brain voxels were computed and a whole brain connectivity matrix for each participant was constructed. The individual correlation matrices were then transformed to a z-score matrix using a Fisher r-to-z transformation. For a given voxel, FCS was computed as the sum of the z-values between the given voxel and all other voxels. We restricted our analyses to correlations above a threshold of r = 0.1 to eliminate weak correlations possibly arising from noises. These FCS maps were smoothed with a 4 mm full-width at a half-maximum (FWHM) Gaussian kernel [20, 31].

### Statistical analysis

The χ2 test was used to compare the sex differences, and one-way analysis of variance (ANOVA) was used to check for differences in age, educational years and SPQ among the three genotype groups (AA, AG and GG). Although there is study has suggested a recessive effect (AA) of rs204993, there is no consensus about analytically grouping the genotypes. Hence, we performed with an unbiased approach that of three possible effects were all considered: additive (GG versus GA versus AA genotypes), dominant (A allele carriers versus GG); recessive (AA versus G allele carriers). For additive effect, one-way ANOVA was performed to determine the changed regions of FCS for healthy participants among the three genotype groups. For dominant or recessive effect, two sample t-tests were performed (A allele carriers versus GG, AA versus G allele carriers). There tests were constrained in a gray matter mask, and multiple comparison corrections were based on a Gaussian random field theory (voxel-level *p* < 0.001; cluster-level *p* < 0.05). The FCS value of the changed regions was extracted and compared with one-way ANOVA among the three genotype groups, and post hoc analysis was conducted using the LSD method. Spearman’s correlation analyses were performed to assess the correlation between the values of FCS and the behavior scores (total SPQ scores and its sub-domains) for each *NOTCH4* genotype group (the significance level: *p* < 0.05/5, two-tail).

## Results

### Demographic information and the genetic effect on behavior performance

Table 1 shows the demographic, genetic, and behavioral data for participants included in the final analysis. The genotype distribution of *NOTCH4* rs204993 in the current study (AA = 142, AG = 188, and GG = 72) was consistent with a previous report of variations of this gene in the healthy Chinese Han population [17]. Genotype groups did not significantly deviate from Hardy-Weinberg equilibrium (χ2 = 0.505, *p* = 0.477). There was no significant difference in sex, educational years, and age between the genotype groups. There was also no significant difference of SPQ between the genotype groups.

**Table 1:** Demographic and behavioral-neuroimgaing information for participants grouped by the rs204993 genotype (mean ± SD)

	AA	AG	GG	Statistics	*P* value
Number of participants	142	188	72	/	/
Sex (male/female)	68/74	88/100	41/31	2.250	0.325
Age (years)	20.761 ± 1.104	20.942 ± 1.061	21.097 ± 1.115	2.490	0.084
SPQ	26.930 ± 13.853	26.553 ± 14.038	31.167 ± 14.470	2.955	0.051
FCS in occipital cortex	0.379 ± 0.461	0.612 ± 0.531	0.755 ± 0.542	15.354	**< 0.001**

*SPQ* schizotypal personality questionnaire, *FCS* functional connectivity strength, *SD* standard deviation

### The genetic effect of rs204993 on FCS

**Fig. 1:**
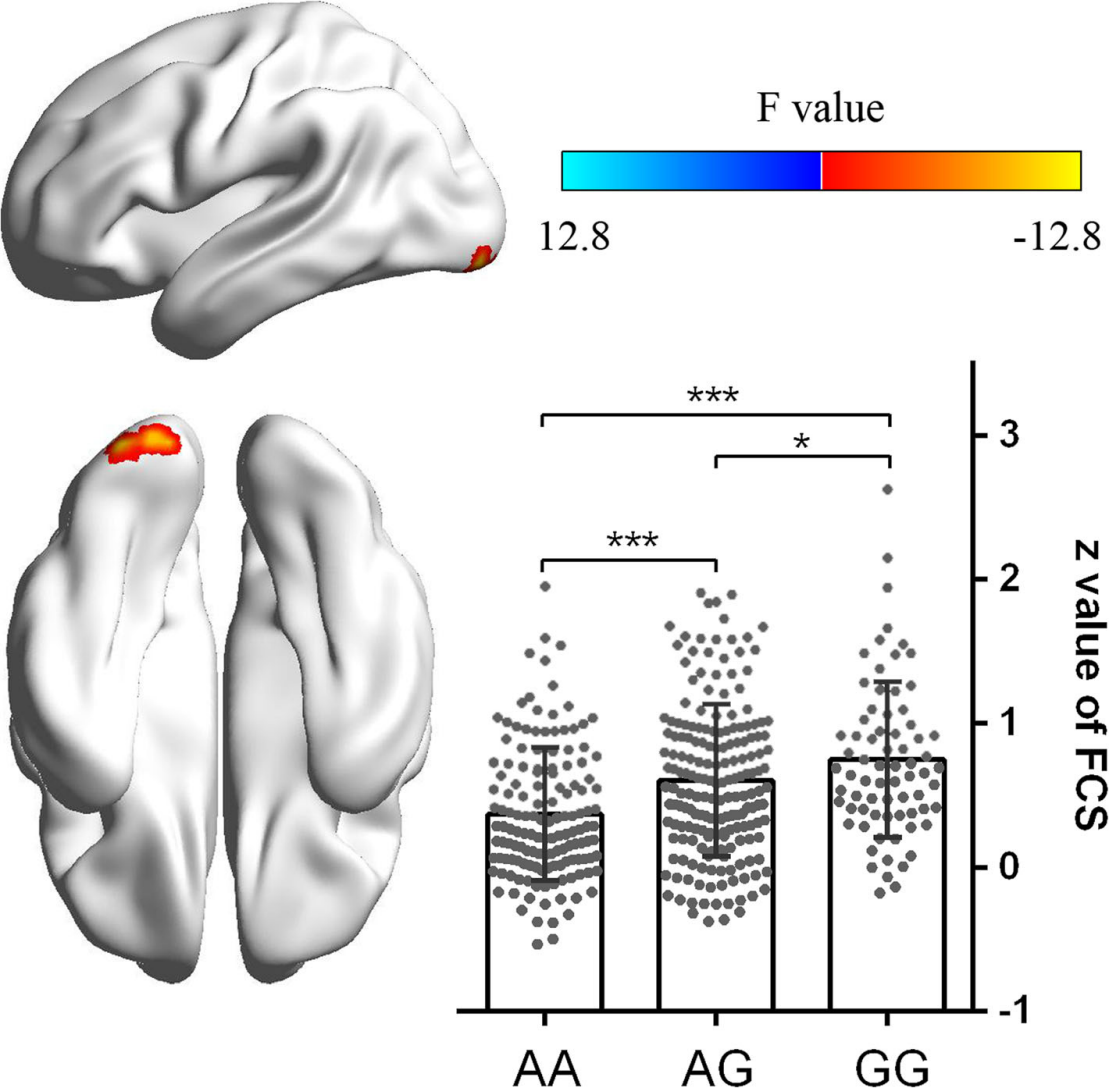
Modulatory effect of rs204993 on the functional strength (FCS). The X-axis shows the different genotypes, and the Y-axis shows the mean ± SD of the FCS in the occipital cortex. Group comparisons revealed that the FCS in the occipital cortex was the highest in GG individuals and decreased from GG and AG individuals to AA individuals. All thresholds for comparisons were set as a whole brain Gaussian random field (GRF) correction (voxel *P* < 0.001, cluster *P* < 0.05)

After controlling for demographic factors, only the additive effect (GG versus GA versus AA genotypes) was survived after corrected using Gaussian random field theory (voxel-level *p* < 0.001; cluster-level *p* < 0.05). The results showed that FCS was significantly abnormal in the left occipital cortex among the *NOTCH4* genotypes (Fig. 1). The FCS values of the above cluster were then extracted in the three genotype groups. We found a main effect of the genotype on FCS values (*F* = 15.354, *p* < 0.001). Post hoc analysis showed AA individuals having reduced values when compared with AG and GG participants (*p* < 0.001 for both) and AG individuals having reduced values when compared with GG participants(*p* = 0.05) (Fig. 1 and Table 1). No cluster was survived after GRF multiple comparison corrections based dominant or recessive statistical model.

### Correlation analyses between SPQ and FCS

**Fig. 2:**
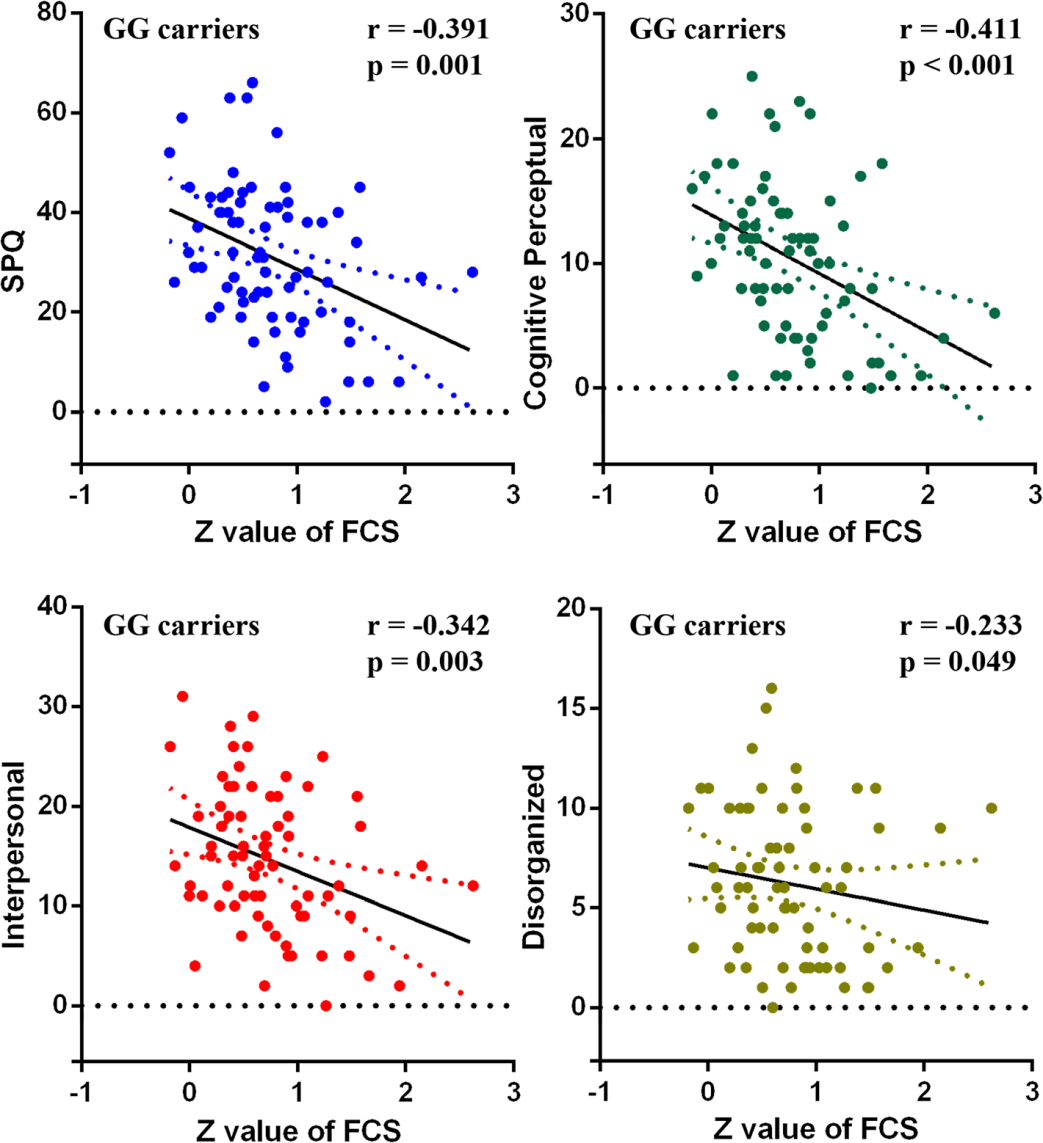
Relationship between the functional strength (FCS) in the occipital cortex and schizotypal traits among the GG genotype carriers. The FCS in the occipital cortex was negatively correlated with the score of schizotypal personality questionnaire (SPQ) and its subscale scores (cognitive perceptual and interpersonal) among the GG genotype carriers). Note: ^***^p < 0.001, ^*^p < 0.05

**Fig. 3:**
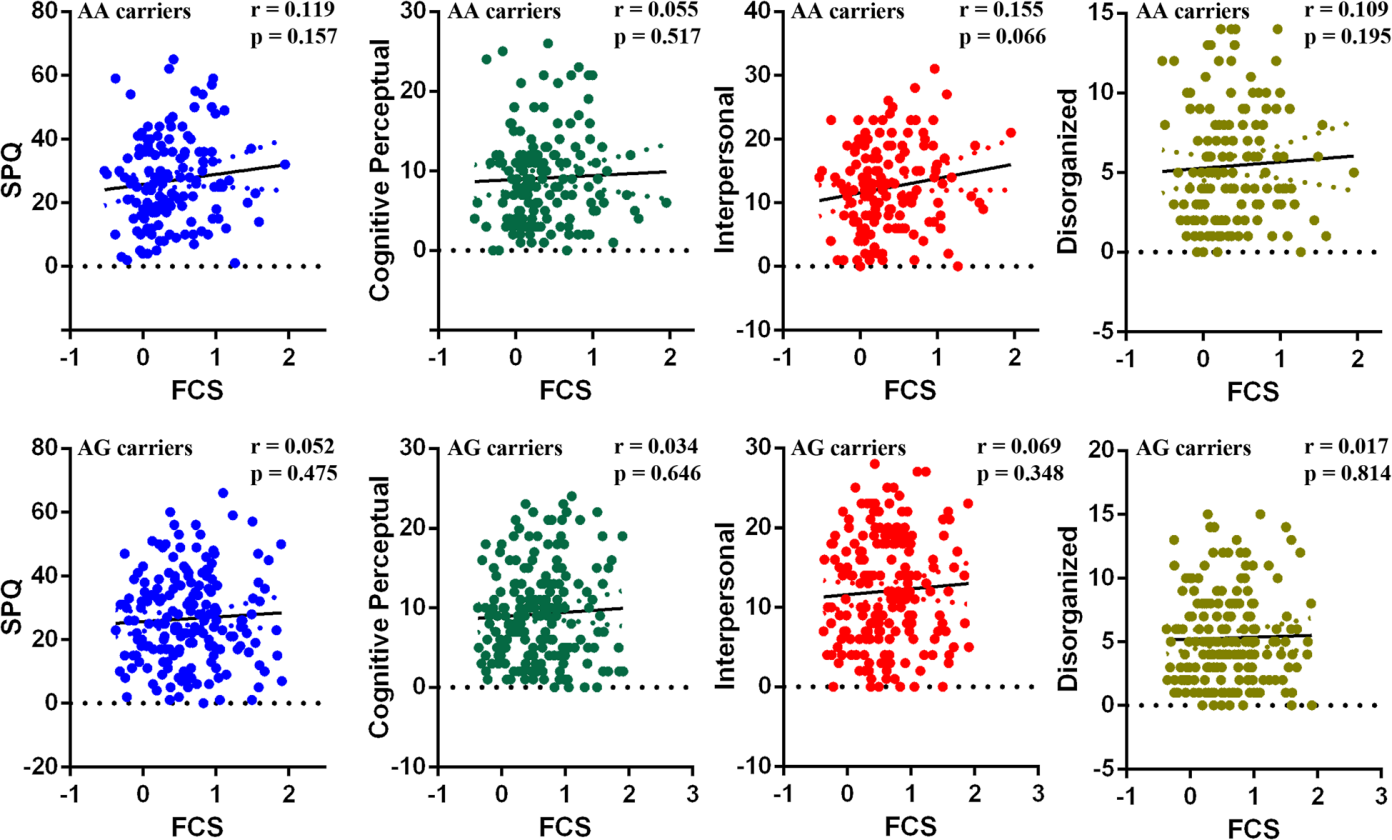
Relationship between the functional strength (FCS) in the occipital cortex and schizotypal traits among the AA and AG genotype carriers. No significant correlation was found between the FCS in the occipital cortex and the schizotypal personality questionnaire scores or its subscale scores

The FCS values in the left occipital cortex were negatively associated with the total SPQ scores (*r* = − 0.391, *p* = 0.001), cognitive-perceptual scores (*r* = − 0.411, *p* < 0.001), interpersonal scores (*r* = − 0.342, *p* = 0.003) and disorganized scores (*r* = − 0.233, *p* = 0.049) among GG participants (Fig. 2). No significant relationship was found between the values of FCS and the behavior scores (total SPQ scores and its sub-domains) for the AA or AG group (Fig. 3).

## Discussion

In the current study, we aimed to investigate the genetic effect of the schizophrenia-related gene (*NOTCH4* rs204993 genotype) on brain function and its relationship with schizotypal traits among healthy volunteers. Using a large sample size, we found that functional connectivity in the occipital cortex was the highest in GG individuals and decreased from GG and AG individuals to AA individuals. Notably, the values of FCS in the left occipital cortex were negatively correlated with total SPQ scores and its subset scores (cognitive-perceptual and interpersonal scores) in GG individuals.

Previous studies have reported that *NOTCH4* polymorphism of rs204993 is closely related to schizophrenia [12, 13]. However, as a candidate gene for schizophrenia, we did not find a direct effect of *NOTCH4* rs204993 on the schizotypal traits among healthy volunteers. Even so, it was notable that we found the modulatory effect of *NOTCH4* rs204993 on brain function (the functional connectivity of occipital cortex), which was associated with schizotypal traits. The impact of the *NOTCH4* gene on the central nervous system has been consistently reported [32]. The *NOTCH4* gene is one member of the *NOTCH* family, which may code for the notch protein, which was identified as a Drosophila neurogenic protein during embryogenesis [33]. Functionally, the *NOTCH* family mainly controls the cell fate in the process of neurodevelopment, which promotes proliferative signaling [34, 35]. Therefore, the *NOTCH4* gene variants may potentially affect the function or expression level of the Notch4 protein, which in turn influences the neurodevelopment of certain psychiatric disorders, such as schizophrenia and schizotypal traits. In the present study, we found an aberrant functional connectivity in the left occipital cortex among carriers with the *NOTCH4* rs204993 schizophrenia-risk genotypes.

The occipital cortex plays an important role in the neural circuits of perceptual processing, such as visual and auditory processing [36, 37]. Considerable studies have confirmed that schizophrenia patients and their unaffected siblings present with impaired perceptual performance, which is associated with impaired structural and functional connectivity in the occipital cortex [38–40]. Furthermore, the occipital lobe has also been gradually recognized as the neural basis for perceiving personal communication and social interactions [41–43]. Of course, some studies have shown that negative symptoms in patients with schizophrenia are associated with abnormal changes in the occipital cortex [44–46]. For example, abnormal connectivity between the occipital cortex and ventral tegmental area is related to the negative symptoms in schizophrenia [44]. In general, the occipital cortex may be a common neural pathway for the processing of positive and negative symptoms in schizophrenia. Consistent with this possibility, our study showed that the occipital-cortex functional connectivity was related with cognitive-perceptual and interpersonal subscores, which represented the positive and negative symptoms in schizotypal traits. We therefore speculate that the *NOTCH4* gene may induce the occurrence and development of diseases by affecting the neurodevelopment of occipital cortex.

It is worth noting that we found that the FCS of the occipital cortex was degressive in sequences of the GG, AG, and AA genotype groups that suggest an additive effect of rs204993. This is not in complete agreement with a recessive effect revealed by the previous results that the AA genotype of rs204993 is associated with a higher risk for schizophrenia in the Chinese Han population [17]. The difference between schizophrenia and schizotypal traits may explain this discrepancy. As our results suggested, individuals with the AG genotype also presented lower FCS in the occipital cortex, when compared with the GG genotype carriers, which may indicate the risk of schizotypal traits in the AG genotype carriers, but not the risk of schizophrenia.

It is interesting that the correlation between occipital-cortex function and SPQ is restricted to specific genotype. Detailedly, individual occipital-cortex FCS was inversely correlated with individual SPQ and its sub-domains (cognitive-perceptual and interpersonal scores) only for GG carriers who may present lower risk for schizophrenia. These results implicate a modulation of occipital-cortex function with cognitive-perceptual and interpersonal ability that the individuals with higher occipital-cortex FCS present more normal cognitive-perceptual and interpersonal ability, which are featured impairments in schizophrenia. The disappearance of such correlation may suggest an impaired modulation of occipital-cortex function with cognitive-perceptual and interpersonal ability in among relative high-risk individuals (AA and AG carriers). Similarly, this genotype-depended correlation has been reported by previous genetic imaging study [47]. Another interesting finding of the present study was that the significant difference among groups was only located at the left occipital cortex and not the right. We speculate that the left-hemisphere dominant is the potential factor underlying this lateralization. Although we did not collect the handedness data, based on a survey reported about 87.7–94.8% right hand domination in Chinese population [48], we believe that most participants in present study are left-hemisphere dominant. The left occipital cortex may prefer to involve in mental and emotional processing, hence, left occipital-cortex connective decline may more contribute to schizophrenia symptoms.

Several potential limitations of our study need to be considered. First, we did not find a direct modulatory effect of the *NOTCH4* gene on schizotypal traits (no significant difference between *NOTCH4* genotype groups). The gross feature of the schizotypal-trait assessment (SPQ) may partly explain this defect. Second, the absence of long scanning durations reduced the reliability of measuring functional connectivity. Third, our study did not reveal the cognitive mental mechanism of *NOTCH4* gene modulating the occipital cortex and its relationship with schizotypal traits. Fourth, previous studies confirmed the relationship between schizophrenia and NOTCH4 polymorphisms, including single nucleotide polymorphisms (SNPs) (e.g., rs520692, rs3131296, rs204993, and rs2071287). But we only explored the relationship between FCS of left occipital cortex and the NOTCH4 rs204993 genotype in populations with schizotypal traits. Further studies are needed to analyze the associations with other SNPs of the *NOTCH4* gene. Finally, we did not characterize the modulatory effect of the *NOTCH4* gene on local and long-range functional connectivities, respectively, which may represent distinct biological differences.

## Conclusion

Our results suggested that the common variant in the *NOTCH4* gene, rs204993, modulates the function of the occipital cortex, which may contribute to schizotypal traits. These findings provide insight for genetic effects on schizotypal traits and its potential neural substrate.

## Data Availability

Genetic data in this article are deposited in the NCBI SRA database (accession: SRP268121). Requests to access the MRI data should be addressed to wangkai1964@126.com.

## Abbreviations

FCS: Functional connectivity strength
SPQ: Schizotypal Personality Questionnaire
rs-fMRI: Resting-state functional magnetic resonance image
*NOTCH4*: Neurogenic locus notch homolog protein4
MHC: Major histocompatibility complex
SNPs: Single nucleotide polymorphisms
DPARSF: Data Processing Assistant for Resting-State Functional
SPM8: Statistical parametric mapping software package
REST: Resting State Functional MR Imaging Toolkit
FWHM: Full-width at half-maximum
